# Evaluation of the tear parameters of ovulation induction patients in a short time period with anterior segment optical coherence tomography

**DOI:** 10.5935/0004-2749.20200095

**Published:** 2024-02-11

**Authors:** Eser Çolak, Mahmut Oğuz Ulusoy, Mehmet Ufuk Ceran, Ümit Taşdemir, Ali Kal, Emel Ebru Özçimen

**Affiliations:** 1 Obstetrics and Gynaecology Department, Faculty of Medicine, Baskent University, Konya, Turkey; 2 Ophthalmology Department, Faculty of Medicine, Baskent University, Konya, Turkey

**Keywords:** Estradiol, Dry eye syndrome, Fertile period, Menopause, Tomography, optical coherence, Estradiol, Síndromes do olho seco, Periodo fertil, Menopausa, Tomografia de coerência óptica

## Abstract

**Purpose:**

The effects of sex steroid hormones on tearparameters are known. Theaim of this studywas to examine the effects on tear parameters during exposure to high-dose sex steroids in a short period of time.

**Methods:**

Forty patients who were admitted to the infertility clinic of our hospital and planned to undergo ovulation induction with exogenous gonadotropins were included in our study. Prior tothe initiation of ovulation induction, the basal levels of estradiol were measured on day 3 of the menstrual cycle and ophthalmologic examinations were performed by the ophthalmology department of our hospital. The estradiol levels were-measured on the day ofovulation induction usinghuman chorionic gonadotropin and compared with basal estra diol; eye examinations were also repeated.

**Result:**

Forty women with reproductive period and average age of 33.3 ± 4.2 years were included in this study. Basal levels of estradiol were significantly (p<0.001) higher after ovulation induction than before induction. The scores in the break-up timeand after induction were 6.2 ± 2.8 sn and 8.4 ± 1.4 sn, respectively. The values of Schirmer’s test were 14.3 ± 7.1 mm and 20.6 ± 6.2 mm before and after induction, respectively. Both values were significantly higher after ovulation induction (p<0.001; p=0.001, respectively).

**Conclusion:**

We observed improvemet in tear function tests following the use of estradiol even for a limited time.The use of estradiol during menopause may improve dry eye symptoms in patients.

## Abstract (pt)

**Objetivo:**

Os efeitos dos hormônios esteróides sexuais nos parâmetros lacrimais são conhecidos. O objetivo deste estudo foi examinar como os efeitos nos parâmetros lacrimais durante a exposição a altas doses de esteróides sexuais em um curto período de tempo.

**Métodos:**

Quarenta pacientes que foram admitidas na clínica de infertilidade do nosso hospital e planejavam a indução de ovulação por gonadotropinas exógenas. Antes do início da indução da ovulação, os níveis basais de estradiol foram medidos no terceiro dia do ciclo menstrual e os exames oftalmológicos foram efetuados pelo Departamento de Oftalmologia do nosso hospital. Os níveis de estradiol foram medidos no dia da indução da ovulação usando gonadotrofina coriónica humana e comparados aos estradiol basal; exames oftalmológicos também foram repetidos.

**Resultado:**

Quarenta mulheres com período reprodutivo e idade média de 33,3 ± 4,2 anos foram incluídas neste estudo. Os níveis basais de estradiol foram significativamente maiores (p<0,001) após a indução da ovulação do que antes desta. Os resultados dos testes de ruptura do filme lacrimal e após a indução foi de 6,2 ± 2,8 s e 8,4 ± 1,4 s respectivamente. Os valores do teste de Schirmer foram 14,3 ± 7,1 mm e 20,6 ± 6,2 mm, respectivamente antes e depois da indução. Ambos os valores foram significativamente maiores após a indução da ovulação (p<0,001; p=0,001 respectivamente).

**Conclusão:**

Observamos uma melhora nos testes de função lacrimal após o uso de estradiol, mesmo por tempo limitado. O uso de estradiol durante a menopausa poderá melhorar os sintomas do olho seco em pacientes.

## INTRODUCTION

Dry eye disease (DED) is a problem especially for older women. According to the 2007 dry eye workshop, DED is a multifactorial disease involving tear components and the ocular surface. This disease is accompanied by increased osmolarity of the tear film and inflammation of the ocular surface^(1)^. Dry eye adversely affects the quality of life by compromising activities, such as reading, watching television, using computers, and driving. Other manifestations include itching, burning, and decreased visual acuity^(2)^. The currently available treatments for DED are inadequate, and the condition has become a growing public health problem^(3)^.

A healthy tear film consists of three main components, namely mucin, lipid, and andaqueous. There are inflammation detection receptors formed by chemical and mechanical irritant substances in the eye. Following the stimulation of these receptors, the production of tears from the lacrimal glands is mediated by the autonomic nervous system^(4)^. Lacrimal glands are the main sources of the aqueous layer of the tear film^(5)^. Peroxidases are mostly antimicrobial and antioxidant enzymes found in exocrine secretions, such as saliva and tears^(6)^. Plasma 17b-estradiol (E2) levels and peroxidase activity have been positively correlated with the menstrual cycles of women in the reproductive period^(7)^. In addition, lactoperoxidase activity in the lacrimal fluid was significantly decreased in menopausal patients. This decreasechangesthe tear protein content and causes DED^(8)^.

The secertion of mucin from the goblet cells is necessary for the protection of the conjunctival thickness and moisture in the eyes. Correlated with changes in vaginal mucosa, the epithelium thickness in the conjunctiva varies with the menstrual cycle. It has been shown that the epithelium is thicker in the late follicular phase, which exhibits the highest levels of estrogen^(9)^.

The lipid layer is important for the stabilization of the tear film. Lipid is mainly produced from the meibomian glands, which are responsible for reducing surface tension and preventing tears. The main event causing thinning of the lipid layer is the clogging of these glands and decrease of secretion^(10)^. The production and secretion of the meibum by the meibomian glands is influenced by hormonal, neural, and mechanical factors. Both androgens and estrogens regulate secretions by the meibomian glands. It is established that androgens increase lipid synthesis from the meibomian glands. However, the effects of E2 on lipid synthesis and catabolism are controversial^(11)^.

The different levels of sex hormones in the plasma cause changes in many tear components, and the anatomical and functional structure of the ocular surface^(12)^. Sex hormone dysfunction causes progression of disease and resistance to treatment in DED and vernal keratoconjunctivitis, which are two major diseases of the eye^(11)^. In cases of premature ovarian failure, autoimmune diseases, and menopause, dysfunction occurs in the meibomian glands, especially due to androgen deficiency. This leads toa negative effect on the lipid tissue of the eye^(13^,^14)^. Dry eye symptoms are increased in women receiving aromatase inhibitors as adjuvant or prophylactic treatment for breast cancer^(15)^. There is a decrease in the free E2 levels due to the increased binding of globulin by sex hormones during pregnancy. There is also an increa se in the levels of progesterone and prolactin. Lacrimal gland secretion and inflammation of the ocular surface during the pregnancy period have been attributed to these hormonal changes^(16)^.

There are numerous studies investigating the effects of sex hormones on tear function during the postmenopausal and perimenopausal period. However, there are no studies examining these effects in the reproductive age group. Therefore, the effects of E2 on these functions remain unclear. The aim of this study was to observe the changes occurring in the reproductive age group.

## METHODS

### Study design and population

This study was performed between June 2018 and Ja nuary 2019, and approved by the local ethics committee of Baskent University (Konya, Turkey) (registration number KA09/184). The study adhered to the tenets of the Declaration of Helsinki and written informed consent was provided by all participants.

Forty patients who were admitted to the infertility clinic of our hospital and planned to undergo ovulation induction with exogenous gonadotropins were included in our study. Prior to the initiation of ovulation induction, the basal levels of E2 were measured on day 3 of the menstrual cycle, and ophthalmologic examinations were performed by the ophthalmology department of our hospital. The E2 levels were-measured on the day of ovulation induction with human chorionic gonadotropin (hCG); eye examinations were performed and values obtained before and after induction were compared. Exclusion criteria were a history of a primary condition that could cause dry eye (e.g., pterygium, dellen, previous refractive surgery), any systemic disease that could effect measurements, systemic connective tissue disease, a history of any significant ocular surface disea se, ocular inflammation, or other ocular surgery within the past year, use of a contact lens during the previous month, use of eye medications or artificial tears during the previous month, and presence of another systemic diseases affecting the eye (e.g., diabetes, hypertension, autoimmune disease, and connective tissue disease). In addition, patients with elevated levels of progesterone during ovulation induction were excluded.

### Ovulation induction

Basal E2 levels were measured on the third day of the menstrual cycle and the follicles were evaluated through transvaginal ultrasound. Standard recombinant follicle stimulating hormone (Puregon, Merck Sharp and Dohme, The Netherlands) was administered at a daily fixed dose of 150-225 IU for controlled ovarian hyperstimulation. Follow-up scans were performed every 2-3 days thereafter. Human menopausal gonadotropin (Menopur; Ferring) was added, as required. According to the protocol, gonadotropin-releasing hormoneantagonist cetrorelix acetate (cetrotide) (0.25 mg per day subcutaneously) was added to the treatment when the dominant follicle was >14 mm or the E2 levels measured in the blood were>300 pg/ml. When at least two leading follicles reached 18-19 mm in diameter, induction of final oocyte maturation was triggered by 6,500 IU of recombinant hCG (Ovidrel; Merck Serono Biopharma) and the levels of E2 were-measured.

### Ophthalmologic examinations

Ophthalmologic examinations were performed prior to ovulation induction and on the day of hCG administration (approximately day 15). The study patients completed the Ocular Surface Disease Index (OSDI) at the beginning of their visit. The OSDI, developed by the Outcomes Research Group at Allergan Inc. (Irvine, CA, USA), is a 12-item questionnaire designed to provide a rapid assessment of the symptoms of ocular irritation consistent with DED and their impact on vision-related functioning. The presence of symptoms during the last week is rated per item using a 5-point scale (0-4) from ‘‘none of the time’’ to ‘‘all of the time’’. The OSDI total score (ranging 0-100) can be calculated with a formula using the sum score of all completed questions^(17)^.

**Figure 1:**
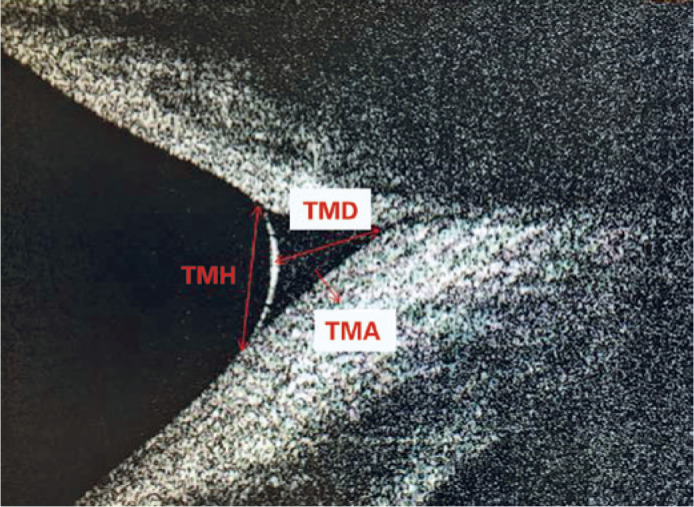
Anterior segment optical coherence tomography image of tear meniscus parameters.

A spectral domain optical coherence system (RTVue-100; Optovue, Fremont, CA, USA) with a corneal adaptor module was used. This system has a 6-mm vertical beam that performs 26,000 axial scans per s and has a 5-mm axial resolution to a depth of 2.8 mm. Vertical images were recorded at the 6-o’clock position of the cornea 3 s after each blink, which was repeated thrice. A built-in caliper was used to measure the tear meniscus height (TMH), tear meniscus depth (TMD), and tear meniscus area (TMA). The mean of the three measurements was used for analysis. TMH was determined as the length from the point where the meniscus intersected with the cornea superiorly to the eyelid inferiorly. TMD was determined as the length from the apex of the fornix to the surface of the tear meniscus, perpendicular to the TMH. The borders of the tear meniscus were marked with a caliper, and integrated analysis software calculated the area in mm^2^ to measure the TMA. Only measurements of the right eye were used for statistical analysis (Figure 1).

(TMA= tear meniscus area; TMD= tear meniscus depth; TMH= tear meniscus height).

Subsequently, conventional dry eye tests were performed, including break-up time (BUT) after fluorescein solution instillation and the Schirmer’s test. Schirmer’s test was performed for a duration of 5 min after instillation of topical anesthetic drops (0.5% proxymetacaine; Alcaine). The filter paper strip was placed in the middle and lateral thirds of the lower eyelid.

### Statistical analysis

Statistical data were analyzed using the SPSS version 21.0 (IBM Corp., Armonk, NY, USA). Values were expressed as the mean ± standard deviation. The normality of the values was analyzed using using the Kolmogorov-Smirnov test. Paired t-test was used according to the Kolmogorov-Smirnov test results. Differences were considered significant at p<0.05. Correlations between the variables were investigated based on the Pearson’s or Spearman’s correlation coefficient and linear regression analyses.

## RESULTS

**Figure 2:**
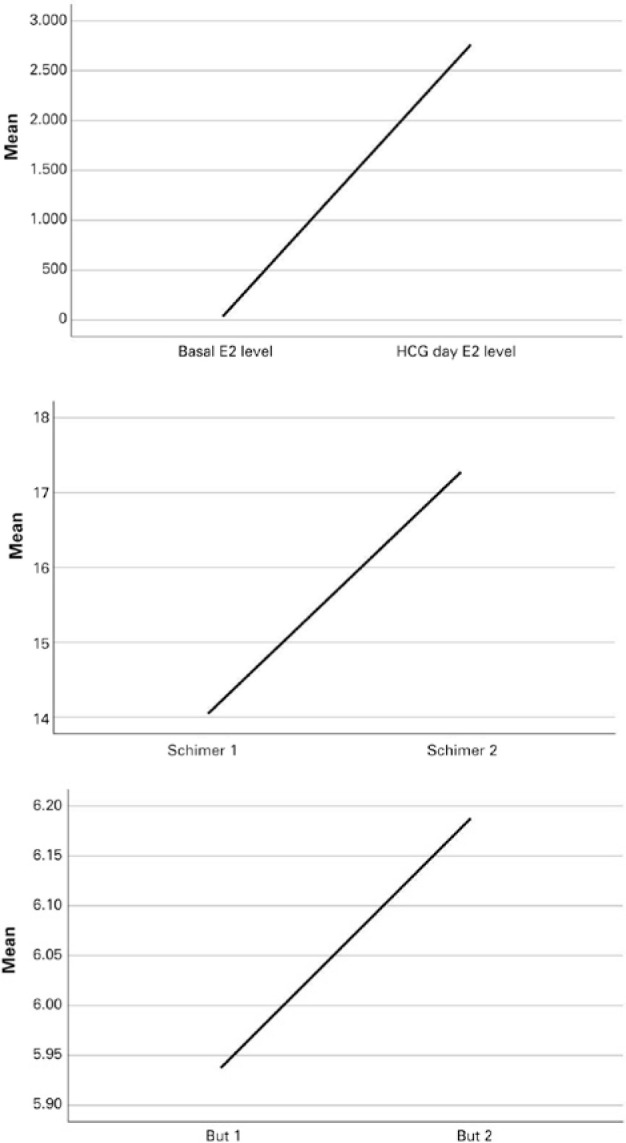
Changes in estradiol, BUT, and Schirmer’s test.

The mean age of the patients was 33.3 ± 4.2 years. The hormonal and tear parameters profilesare presented in table 1. The mean levels of E2 in the serum before and after induction were 37.15 ± 21.6 pg/dl and 2,760.9±1,606.9 pg/dl, respectively. The levels of E2 were significantly higher (p<0.001) after ovulation induction than before induction. There were no significant differences between the values of progesterone, luteinizing hormone, and testosterone (p=0.18, p=0.34, and p=0.82, respectively). The BUT score before and after induction was 6.2 ± 2.8 sn and 8.4 ± 1.4 sn, respectively. The values of the Schirmer’s test were 14.3 ± 7.1 mm and 20.6 ± 6.2 mm, respectively. Both values were significantly higher after ovulation induction (p<0.001, p=0.001, respectively) (Figure 2). The OSDI scores were 21.1 ± 17.6 and 20.5 ± 17.1, respectively, and there were no significant differences (p=0.875). Moreover, there were no significant difference between the scores of TMA, TMD, and TMH (p=0.68, p=0.17, p=0.16, respectively).

**Table 1:** Comparison ofvalues before and after induction

Variable	Before	After	p-value
E2 **(pg/mL)**	37.15 ± 21.6	2760.9 ± 1606.9	**<0.001**
Testosterone **(nmol/L)**	0.88 ± 0.14	0.87 ± 0.16	0.82
P **(ng/mL)**	0.3 ± 0.13	0.33 ± 0.08	0.18
LH **(mIU/mL)**	2.91 ± 1.08	3.14 ± 1.33	0.34
OSDI	21.1 ± 17.6	20.5 ± 17.1	0.87
Schirmer’s test **(mm)**	14.3 ± 7.1	20.6 ± 6.2	**<0.001**
BUT **(sec)**	6.2 ± 2.8	8.4 ± 1.4	**0.001**
TMA (µ**m)**	0.045 ± 0.07	0.051 ± 0.06	0.68
TMD (µ**m)**	187.4 ± 92.1	207.1 ± 94.6	0.17
TMH **(mm**^^2^^)	245.1 ± 133.5	276.6 ± 135.2	0.16

OSDI= ocular surface disease index; BUT= break-up time; TMA= tear meniscus area; TMD= tear meniscus depth; TMH= tear meniscus height; P= progesterone; LH= luteinizing hormone; E2= estradiol.

## DISCUSSION

In this study, we investigated the acute effects of increased E2 levels on tear function. It is established that the hormonal profile is associated with some eye diseases^(12)^. Numerous studies have examined the relationship between hormonal changes and tear functions. However, the effects of these changes on tear func tions and the diseases with which they are associated remain unknown.

There are numerous contrasting studies showing increased and decreased tear functions and secretions following the usage of E2 and other sex steroids. However, all these studies involved patients in the perimenopausal period.In a study, common eye diseases observed in the postmenopausal period were examined and hormone replacement therapy (HRT) exerted a protective effect against dry eye^(18)^. Taner et al. investigated the eye function of 70 postmenopausal patients receiving different HRT. They reported animprovement in the only-tibolone-treated group; however, there were no changes observed in the only estrogenor estrogen + progesterone-treated groups^(19)^. In a similar study, all HRT regimens improved the results of tear function tests and there was no statistically significant difference between treatments^(20)^. However, in another study, treatment with tibolone did not have an effect on the eye; however, treatment with E2 increased the frequency of DED^(21)^.

The frequency of DED in postmenopausal patients who received E2 therapy was lower than that noted in premenopausal patients. In the reproductive age group, patients with ovulation inhibition reported more frequent DED than those with a spontaneous menstrual cycle^(22)^. Similar to our study in terms of the age group, this study showed that sex steroids exert protective effects on ocular surface in the reproductive age group. We have shown that high levels of E2 play a positive role on tear function tests in the premenopausal reproductive age group.

Some studies have shown that the levels of E2 or HRT are not associated with tear function in the premenopausal and postmenopausal period, and only the levels of androgen are linked to a significant improvement inDED^(23^,^24)^. In similar studies, it was reported that different HRT protocols did not improve the tear function of patients, and were associated with risk of dry eye development during long-term treatment^(25^,^26)^.

In a study conducted by Coskuer et al., combined treatment with E2 and progesterone was evaluated in 34 postmenopausal patients. The OSDI scores were decreased after 6 months of treatment, where as the values of Schirmer’s and BUT tests were increased^(27)^. In our study, only the effect of isolated E2 levels was investigated and patients with high progesterone levels were not evaluated. We achieved better score in BUT and Schirmer’s tests at high E2 levels; however, we did not record significant differences in the OSDI test. The reason for the stability of the OSDI test may be the short usage period of the medication. Changes in tear parameters may require a longer period time to show clinical manifestations.

In some studies performed at the cytological level, the number of goblet cells was increased after 3 months of treatment with E2 and tear function tests were improved in postmenopausal patients^(28)^.

Peroxidases in exocrine glands are associated with the levels of E2. Some studies have shown that there is no estrogen receptor in the lacrimal glands; however, the mRNA of the receptor was detected. Therefore, it has been reported that estrogen exerts a protective effect on the eye only through peroxidases^(29)^. A similar study showed increased peroxidase activity and protein secretion following treatment with estrogen in postmenopausal patients^(30)^.

Limitations of our study include the following: the small number of patients; the short duration of exposure to high estrogen levels during the treatment; the OSDI score as a patient-dependent questionnaire; and the failure to answer the questionnaire during the IVF treatment.

This is the first study to measure the effect of estrogen on eye functions in the reproductive period. As a result, the effects of estrogen on the tear parameters in the postmenopausal and perimenopausal periods are controversial. In our study, high levels of estrogen in the reproductive age group exerted positive effects on tear parameters.
